# Systemic delivery and SPECT/CT *in vivo* imaging of ^125^I-labelled oncolytic adenoviral mutants in models of pancreatic cancer

**DOI:** 10.1038/s41598-019-49150-9

**Published:** 2019-09-06

**Authors:** Y. K. Stella Man, Julie Foster, Elisabete Carapuça, James A. Davies, Alan L. Parker, Jane Sosabowski, Gunnel Halldén

**Affiliations:** 1 Centre for Molecular Oncology, Barts Cancer Institute, Queen Mary University of London, London, UK; 2 Division of Cancer and Genetics, School of Medicine, Cardiff University, Cardiff, CF14 4XN UK

**Keywords:** Cancer imaging, Targeted therapies

## Abstract

Early phase clinical trials have demonstrated good therapeutic index for oncolytic adenoviruses in patients with solid tumours when administered intratumorally, resulting in local tumour elimination. Entrapment and binding of adenovirus to erythrocytes, blood factors, and neutralising antibodies have prevented efficient systemic delivery and targeting of distant lesions in the clinic. We previously generated the novel replication-selective Ad-3∆-A20T to improve tumour targeting by increasing the viral dose at distant sites. Here, we developed a protocol to directly radiolabel the virus for rapid and sensitive detection by single-photon emitted computed tomography (SPECT/CT) providing a convenient method for determining biodistribution following intravenous administration in murine models. Longitudinal whole-body scans, demonstrated efficient viral uptake in pancreatic Suit-2 and Panc04.03 xenografts with trace amounts of ^125^I-Ad-3∆-A20T up to 48 h after tail vein delivery. Hepatic and splenic radioactivity decreased over time. Analysis of tissues harvested at the end of the study, confirmed potency and selectivity of mutant viruses. Ad-3∆-A20T-treated animals showed higher viral genome copy numbers and E1A gene expression in tumors than in liver and spleen compared to Ad5wt. Our direct radiolabeling approach, allows for immediate screening of novel oncolytic adenoviruses and selection of optimal viral genome alterations to generate improved mutants.

## Introduction

A promising novel strategy to eliminate solid tumors is the development of cancer-selective, replicating (oncolytic) adenoviral mutants. To date, several adenoviral mutants have been evaluated in clinical trials with demonstrated safety, tumor selectivity and efficacy in conjunction with standard of care interventions^1–3^. In most trials, viruses were administered intratumorally. However, there is a need to further develop the viruses for efficient systemic delivery in order to reach deep and distant metastatic lesions. Major obstacles include the high-affinity binding to erythrocytes and blood factors, in addition to rapid elimination by hepatic Kupffer cells that greatly decrease the available dose at tumour sites^4–7^. Much research is aimed at overcoming these hurdles by developing novel mutants with prolonged circulatory half-life to maximise viral concentrations at the tumour sites^1,8–10^. Biodistribution is usually determined in preclinical *in vivo* models by harvesting tissues from numerous animals at multiple time points to quantify tumour and tissue uptake. In the current study, we developed a novel strategy to select for optimal mutants by rapid monitoring of viral distribution in individual live animals over time without the need for tissue harvesting at every time point.

We recently modified our potent replication-selective oncolytic adenovirus Ad∆∆^11^, to generate a novel mutant, Ad-3∆-A20T with attenuated erythrocyte and blood factor binding and specific targeting to pancreatic ductal adenocarcinomas (PDAC)^12^. The de-targeting modifications reduced viral association to human erythrocytes and complement through ablation of viral fibre-binding to the Coxsackie and Adenovirus Receptor (CAR) and Complement Receptor 1 (CR-1)^13^. To further increase tumour-specific uptake, a 20-amino acid integrin-binding peptide (A20; A20FMDV2) was expressed in the viral fibre-knob^12^. This peptide, derived from the foot-and-mouth-disease-virus (FMDV), selectively binds to αvß6-integrins with high affinity (K_D_ = 0.2nM) via the Arg-Gly-Asp (RGD)-domain^14,15^. The αvß6-integrins are frequently overexpressed in pancreatic, breast and colorectal tumours with negligible expression in normal cells^16–18^. Both Ad-3∆-A20T and the αvß6-integrin targeted wild type virus Ad5A20^19,20^ preferentially infect αvß6-integrin expressing cells while uptake via the typical Ad5-pathway through αvß3- or αvß5-integrins, is significantly less^12^. The deletions in the highly cancer-selective and efficacious Ad∆∆ virus in combination with de- and re-targeting modifications resulted in the generation of the PDAC-selective oncolytic mutant Ad-3∆-A20T, an improved clinical candidate for systemic delivery^12^.

In the current study, we took advantage of the high levels of αvß6-integrins in human PDAC cell lines, and determined viral distribution in xenograft tumours after systemic delivery of the re-targeted and ^125^I-labelled mutants using single photon emission computed tomography (SPECT/CT). Three key areas were investigated in the murine models: (1) the feasibility of radiolabelling adenoviral mutants; (2) the suitability of ^125^I-labelled viruses for imaging and biodistribution studies; and (3) whether this is achievable without the need to conduct any further genetic alterations to the virus. A common, fast and easy method for radiolabelling of proteins involves incorporating ^125^I into Tyr-residues which allow for sensitive imaging detection^21^. However, this method is typically incompatible with biologically active viruses because it is usually performed under non-physiological reaction conditions which include optimal pH and buffer compositions that greatly reduce viral potency^22^. We hypothesised that small animal live imaging technology, SPECT/CT and MRI would rapidly inform on biodistribution and tissue uptake of virus. Imaging in combination with positive detection of viral protein expression and replication/spread at tissue sites would allow for quicker identification of optimal viral mutations. Once identified, these can be further developed into clinically safe and potent therapies. Longitudinal distribution studies often require the sacrifice of multiple animals to analyse harvested tissues at specific time-points and our strategy could potentially provide an economical solution by shortening the time required for screening viral mutants in addition to reducing the number of animals per study. Imaging technology and quantification of radioactively labelled compounds have frequently been used in the discovery and development phases of peptides for example, [^18^F]fluorobenzoyl-A20FMDV2 for αvß6-integrins^17^, ^18^F-, ^11^C- and ^123^I-labelled ligands for the serotonin transporter^23^ and the somatostatin analogue ^111^In-octreotide^24^. Our findings suggest that this strategy can be extended to actively replicating viruses.

The sequential steps described in this study demonstrate a novel approach for allowing the efficient screening of novel replication-selective adenoviral mutants during the preclinical phase of biotherapeutics development *in vivo*. We highlight the positive practicalities of directly labelling adenoviral mutants with ^125^I with retained activity for subsequent SPECT/CT image analysis in small animals. We propose a method that can swiftly inform on whole-body distribution, the location of experimental tumours and potential off-target infection sites. In combination with additional biodistribution and mechanistic studies, this method can provide essential data for safe clinical translation.

## Results

### Optimised ^125^I radiolabelling conditions retains adenoviral functions

**Figure 1:**
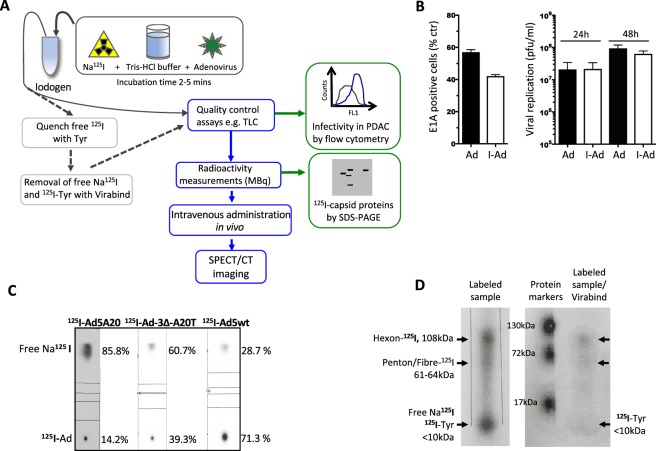
Flow diagram illustrates the labelling of adenoviral mutants with Na^125^I using Iodogen followed by down-stream processing. (**A**) In a typical reaction, 20–27 µl of Tris-HCl (200 mM, pH6.8) was added to an Iodogen tube followed by 25–33.3 µl Na^125^I (85–123 MBq) in 0.04 M NaOH pH11 and incubated for 2 min. This was followed by the addition of 1.23 × 10^8^–2.62 × 10^10^ viral particles (vp) in 200–267 µl Tris-Cl (10 mM pH7.4) corresponding to 7.2 µg–7.65 µg viral proteins. The iodination reaction was terminated after 2.5 min by transferring the mixture to a new tube where it was diluted for *in vivo* imaging. For *in vitro* analytical assays, 10 µl Tyrosine (10 µg) in Tris-Cl pH7.4 was added to quench the reaction, followed by column purification (Virabind). In the final optimised protocol Tris-HCl (200 mM, pH6.8) and 25–33.3 µl Na^125^I (85–123 MBq) in 0.04 M NaOH pH11 were incubated in the Iodogen tube for 2 min and transferred to a new tube containing the virus as above and incubated for another 2.5 min. (**B**) Infectivity and replication in Suit-2 cells comparing non-radioactive labelled I-Ad-3∆-A20T (I-Ad) and mock-infected non-labelled Ad-3∆-A20T (Ad). The reaction conditions were identical to those used for the radioactive ^125^I-incorporation. Infectivity was determined by a specific mouse anti-E1A antibody and detected by secondary anti-mouse FITC-coupled antibodies 24 h post-infection (100 ppc) by flow cytometry (left panel). Viral replication was determined 24 h and 48 h post infection (100 ppc; per infection) (right panel). Averages ± SD, n = 2. **(C**) Aliquots (1 µl) of the reaction mixtures were analysed by instant thin layer chromatography (iTLC-SG) in 85% MeOH/H_2_O. Typical incorporation of ^125^I into the adenoviral capsid proteins were initially 14% (left TLC) and after optimization of pH and labelling protocols 35–71% (middle and right TLC). (**D**) Aliquots (10 µl) of the reaction mixture were analysed on a 4–12% denaturing polyacrylamide gradient gel (SDS-PAGE) and viral coat proteins were identified using a molecular weight ladder matched to the radiograms. In some studies, the reaction mixture was purified on Virabind^TM^ columns and the eluate containing ^125^I-Ad5 (400 µl) was analysed. The recovery yields of virus after Virabind purification were very low and only used in analytical assays. (**C**,**D**) Representative data.

To efficiently radiolabel adenovirus with ^125^I, we considered the optimal conditions required for the reaction when combining the virus, oxidising agent, and radioisotope. The optimal performance of each of these is dependent on specific buffering conditions at certain pH levels. For example, the activity of Ad5 is optimal under physiological conditions, pH7.2-7.8^25–27^. Outside this range the bioactivity of Ad5 is reduced, primarily due to virion aggregation when pH level is lowered from pH8 to pH4^28^. The radioisotope, Na^125^I is commercially available in the form of NaI/0.04 M NaOH and is stored at pH10-12 to preserve stability whereas the oxidising reagent (Iodogen) operates most efficiently at pH6.5 and is less efficient at higher pH^29,30^. It was therefore necessary to identify the optimal pH at which all three reagents were most compatible to perform the radiolabelling process. An overview of the labelling procedure is summarised in Fig. 1A, which include details of the experimental tests used at the earlier stages of protocol development for optimisation purposes.

We tested various reaction conditions in the absence of radioactivity by replacing Na^125^I in 0.04 M NaOH with non-radioactive NaI in 0.04 M NaOH, to determine the preservation of viral infectivity and replication. The infectivity of I-labelled Ad5A20 (expressing EGFP) in the αvß6-integrin expressing Panc04.03 cells was analysed by flow cytometry (Supplementary Fig. 1A). Viral infection decreased from 42% (positive control) to 15% as a direct consequence of Iodogen exposure, an essential step in the protocol, used to oxidise the NaI prior to virus labelling. A further reduction in infectivity was observed upon addition of NaI/NaOH, due to the alkalinity of NaOH (pH10-12). The addition of 200 mM Tris-HCl (pH6.8 or pH4.0; dependent on batch of Na^125^I) lowered the final pH to 7.2-7.8 during the oxidation step and thereby partially restored viral infectivity (Supplementary Fig. 1B). Moreover, direct exposure of the virus to Iodogen greatly decreased the viral activity. However, transferral of the oxidised Na^125^I from the Iodogen tube to a tube containing only the virus and buffer eliminated viral oxidation. We applied the established optimal conditions (pH7.4-7.8 and 100 mM Tris pH4.0) to evaluate infection and replication of our cancer-selective Ad-3∆-A20T labelled with non-radioactive NaI in cultured Suit-2 cells. Infection with I-Ad-3∆-A20T at 100ppc, resulted in 42 ± 3% of infected cells compared to 58 ± 4% for the non-labelled mock control at 24 h (Fig. 1B; left panel). Comparable levels of replication were observed at 24 h, 2.1 × 10^7^ ± 3.5 × 10^6^ pfu/ml (non-labelled) and 2.2 × 10^7^ ± 0.9 × 10^7^ pfu/ml (labelled) which increased to 1.6 × 10^8^ ± 0.8 × 10^8^ pfu/ml and 6.3 × 10^7^ ± 1.1 × 10^7^ pfu/ml, respectively by 48 h (Fig. 1B; right panel).

Next, we used Na^125^I under the same conditions to evaluate the efficiency of the labelling reaction. The specific activity of free ^125^I versus virus-bound ^125^I was determined by thin layer chromatography (TLC) (Fig. 1C). The three viruses used in this study, Ad5A20, Ad-3∆-A20T, and Ad5wt showed labelling efficiencies of 14.2–71.3% with the lowest specific activity for Ad5A20 (Fig. 1C). We observed a dependency on high virus concentrations for higher levels of radiolabelling, explaining the lower specific activity for Ad5A20 that had the lowest viral stock concentration. The sample was electrophoresed by SDS-PAGE to verify that the labelled material was indeed ^125^I-virus (Fig. 1D). Three discrete radioactive bands were identified with two corresponding to the major adenoviral capsid proteins; hexon (108 kDa), penton (63.3 kDa) and fibre (61.5 kDa) and a third band of ≤10 kDa representing the remaining free ^125^I in the sample and ^125^I-Tyr. To separate labelled virus from non-incorporated ^125^I, column purification was performed. Although pure ^125^I-labelled virus was successfully isolated, the yield was poor (<25% of functional virus) and its use was subsequently discontinued (Fig. 1D; right panel).

In conclusion, the adapted labelling protocol had minimal effects on viral function; infection and replication rates remained largely unaffected. Although, the modified protocol is not optimal for the iodination reaction, sufficient ^125^I for imaging analysis was incorporated in the viral capsid proteins (see below).

### Detection of ^125^I-Ad5A20 in pancreatic αvß6-integrin-expressing Panc04.03 xenografts after systemic delivery

**Figure 2:**
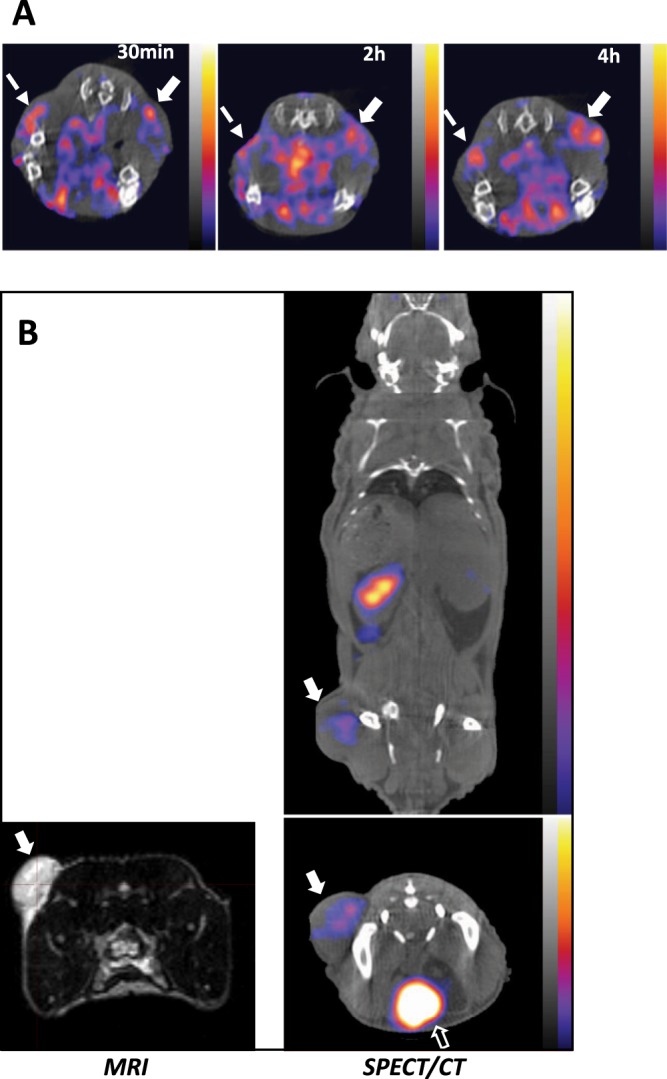
^125^I-Ad5A20 accumulates in Panc04.03 and Suit-2 pancreatic tumour xenografts after tail vein injection and imaged by SPECT/CT. (**A**) One animal with small bilateral subcutaneous and internal Panc04.03 tumours imaged by SPECT/CT. The αvβ6-retargeted, and radioactively labelled ^125^I-Ad5A20 mutant (8.2 × 10^9^ vp/animal; 10.8 MBq/animal) was injected via the tail vein and imaged after 30 min, 2 h and 4 h. White solid arrows indicate subcutaneous tumour; white dashed arrows indicate internal tumour, one representative animal shown, n = 3. (**B**) Left panel; T2-weighted MRI image of one representative animal with a subcutaneous Suit-2 tumour in one flank indicated by white arrow, prior to virus administration. Right panel; Intravenous administration of the αvβ6-retargeted and radioactively labelled ^125^I-Ad5A20 in 200 µl (7 × 10^9^ vp of 2.5 mBq/vp) in the same animal, imaged by SPECT/CT 24 h later. White arrows indicate tumour; white open arrow indicate bladder, one representative animal shown, n = 3. (**A**,**B**) The scale for all SPECT/CT images is 0.006–0.0225 kBq.

We previously determined αvß6-integrin expression in 15 human pancreatic ductal adenocarcinoma (PDAC) cell lines and the highest levels were detected in Panc04.03 cells^12^. Subcutaneous inoculation of Panc04.03 cells in immune-deficient mice formed small tumours (18–50 µl) after ≥3 weeks with the support of Matrigel. Three mice with bilateral tumours were selected for systemic administration of ^125^I-Ad5A20 (8.2 × 10^9^ vp/animal; 10.8 MBq/animal) via tail vein injection and were imaged by SPECT/CT (one representative mouse; Fig. 2A, additional images in Supplementary Fig. 2A). The viral particles of ^125^I-Ad5A20 were detected in tumours at all tested time points (30 min, 2 h, and 4 h after injection), indicating that sufficient amounts had reached the tumour tissue *in vivo*. High uptake of radioactivity was also observed in the liver and gastrointestinal tract that decreased from 2 h onwards, and likely reflected the presence of free ^125^I in the injection mixture (<60% of free ^125^I) in addition to non-specific hepatic elimination of virus and label (Supplementary Fig. 2B). Subsequent image quantification was performed at later time points (24–48 h) to circumvent the initial non-specific abdominal (mainly hepatic) accumulation/clearance of free ^125^I and ^125^I-Ad5A20 allowing for more accurate measurement of tumour-associated radioactivity after imaging.

### Accumulation of ^125^I-Ad5A20 in Suit-2 pancreatic tumour xenografts up to 24h after systemic delivery

We have previously established *in vivo* subcutaneous xenograft models using the human pancreatic αvß6- and αvß5- integrin expressing cancer cell line, Suit-2^12,31^. All subsequent *in vivo* studies were performed using this model since they rapidly and consistently generate reproducible subcutaneous tumours; 100–200 µl in 2–3 weeks (Fig. 2B left; MRI image). ^125^I-Ad5A20 was prepared to 2.5 mBq/vp whereby 39% of ^125^I in the reaction mixture was incorporated into the virus. A total of 7 × 10^9^ vp of ^125^I-Ad5A20 was injected via the tail vein in each mouse (n = 3). The majority of radioactivity was detected in liver and spleen 4 h after administration (Supplementary Fig. 2C) that markedly decreased after 24 h when radioactivity levels were higher in tumours than in hepatic tissue (one representative animal in coronal and transverse views, SPECT/CT images; Fig. 2B right). Whilst the radioactivity associated with the liver, spleen and gastrointestinal tract decreased over time (4–24 h), levels in urine increased (Fig. 2B, additional images in Supplementary Fig. 3A).

Collectively, these studies demonstrate that the retargeted Ad5A20 quickly reached the tumours after systemic delivery and was maintained up to 24 h. Additionally, tumour volumes estimated by the MRI survey scan correlated with end point volume (tumour weight) (160 µl in Fig. 2B), and was similar to calliper measurements (140 µl). Combining calliper measurements with MRI and SPECT/CT imaging is therefore a valuable strategy for determining viral efficacy, internal tumour growth and tissue distribution, respectively, over time.

### Rapid uptake of the ^125^I-labelled oncolytic mutant Ad-3∆-A20T and Adwt in Suit-2 pancreatic tumour xenografts followed by efficient elimination by liver and spleen

**Figure 3:**
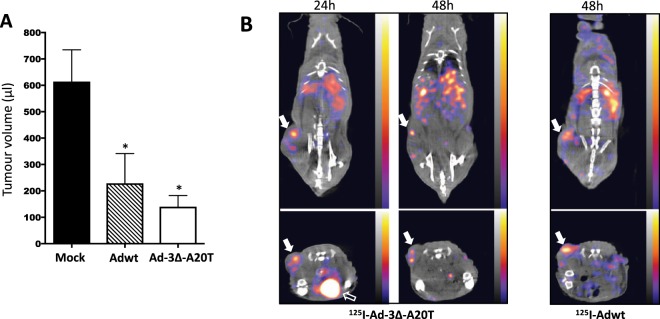
Biodistribution of the novel retargeted oncolytic mutant ^125^I-Ad-3∆-A20T and ^125^I-Ad5A20 after intravenous delivery. (**A**) Suit-2 cells (1 × 10^6^ cells) were inoculated subcutaneously in one flank of CD^*nu/nu*^ athymic mice. When tumours reached 100 µl ± 20 µl unlabelled viruses were administrated intra-tumourally (3 × 10^9^ vp/injection) on day 1, 3 and 6. Tumour growth was determined with caliper instrument on day 18. One-way Anova, Krusaliss Wilkinson post-test *p < 0.01 compared to mock control, 8 animals/group. (**B**) Intravenous administration of ^125^I-Ad-3∆-A20T (1.83 mBq/vp in 200 µl) and ^125^I-Ad5wt (3.27 mBq/vp in 200 µl) via the tail vein in mice with Suit-2 tumours. Live animals were imaged by SPECT/CT after 24 and 48 h, selected images are coronal and transverse views. Tumour uptake was retained up to 48 h (white solid arrows). Open arrow indicates the bladder filled with radioactive metabolites, one representative animal, n = 4. The scale for all SPECT/CT images is 0.00003–0.00015 kBq.

**Figure 4:**
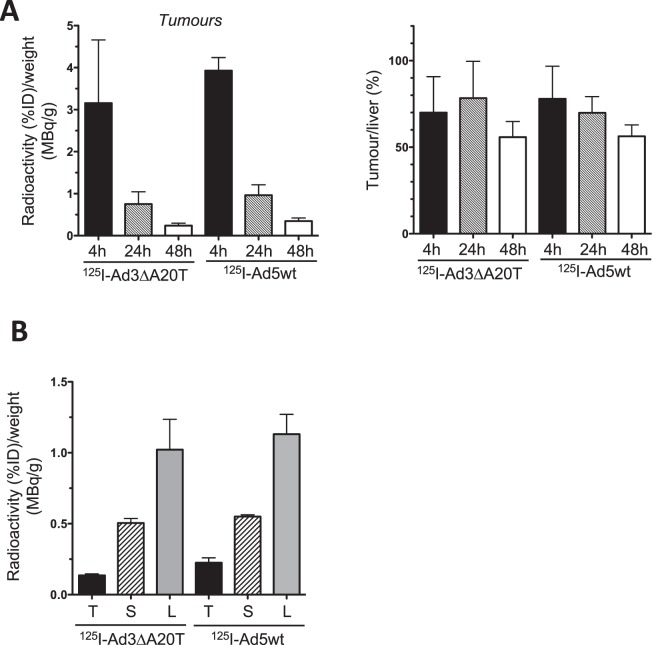
Biodistribution of ^125^I-Ad-3∆-A20T and ^125^I-Ad5A20 quantified using SPECT/CT images after intravenous administration in Suit-2 xenograft models. Tumours, spleen and liver tissues were collected 48 h after treatment of animals with ^125^I-Ad-3∆-A20T and ^125^I-Ad5wt (n = 4 per group). (**A**) Tissues were weighed and correlated to the respective radioactivity SPECT/CT measurement at each time point, determined by ROI analysis using VivoQuant software. Left panel; data were corrected for injected dose (%ID) of radiolabel in tumours at 4, 24, and 48 h, and related to tissue weight at the end of the experiment (48 h; n = 4/group). Right panel; the radioactivity/g tumour was expressed as percentages of radioactivity/g liver at each timepoint (n = 4/group). (**B**) Radioactivity was measured using a gamma counter at the end of the experiment (48 h) and presented as percentages of injected dose (%ID) of labelled virus and relative to tissue weight; n = 3/group.

Direct intratumoral administration of Adwt or our novel, retargeted replication-selective Ad-3∆-A20T mutant caused significant inhibition of tumour growth in Suit-2 xenograft models (18d; Fig. 3A)^12^. The feasibility of intravenous delivery of Ad-3∆-A20T was investigated using ^125^I-labelled virus in conjunction with SPECT/CT imaging. ^125^I-Ad5wt or ^125^I-Ad-3∆-A20T were administered via the tail vein (7 × 10^9^ vp) in animals with Suit-2 xenografts (n = 4/group). Radioactivity was detected in tumours, liver and spleen from 4 to 48 h (^125^I-Ad-3∆-A20T, 24 and 48 h and ^125^I-Adwt, 48 h; Fig. 3B, additional images Supplementary Fig. 3B). After correcting for injected dose (% radioactivity incorporated into virus and total amount administered) and tissue weight at the end of the study, quantification of the SPECT/CT images showed no significant differences in tumour uptake between treatment groups (Fig. 4A; left panel) (Liver; Supplementary Fig. 4). Tumour to liver ratios of radioactivity were also similar at these early time points (Fig. 4A; right panel). These observations were verified by direct measurements of radioactivity in tissues harvested at the end of the study (48 h) (Fig. 4B).

### More potent viral E1A-expression in Suit-2 tumours after systemic delivery of the oncolytic mutant Ad-3∆-A20T than with Adwt

**Figure 5:**
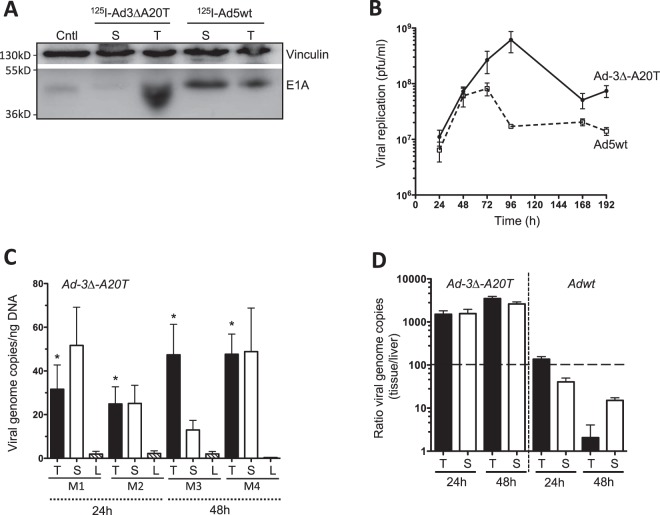
Viral genome amplification in tumour, liver and spleen tissues after systemic administration of Ad-3∆-A20T and Ad5wt. (**A**) Protein lysates were prepared from tumour and spleen tissues harvested from animals treated with viruses (as shown in Fig. 4 with radioactive viruses) at the end of the study (48 h), and used for immunoblotting. The early viral E1A protein was detected with a specific anti-E1A antibody. Vinculin was detected and used as loading control, one representative cropped immunoblot from each treatment group (complete immunoblot in Supplementary Fig. 6). (**B**) Viral replication of Ad-3∆-A20T and Ad5wt over time from 24 h to 192 h, as determined by standard TCID_50_ assay. Averages (pfu/ml) ± SEM, n = 4. (**C**) Copy numbers of viral genomes in tissue specimens harvested from animals 24 h or 48 h after tail vein administration of Ad-3∆-A20T determined by qPCR. Values are expressed as an average copy number of E1A per ng of DNA ± SEM in individual animal; n = 4. Tumour (T), spleen (S), liver (L) tissues, M = mouse. (**D**) Viral genome ratios comparing accumulation in tumour and spleen to liver values for both Ad-3∆-A20T and Ad5wt at 24 and 48 h after tail vein administration. Averages ± SEM, n = 2.

To investigate whether tissue-associated radioactivity correspond to the presence of virus, expression of the early viral gene E1A was determined in tumour and spleen tissues at the end of the study. Interestingly, animals treated with ^125^I-Ad-3∆-A20T presented with higher levels of the E1A protein in tumours, and lower levels in spleen compared to tissues harvested from animals treated with ^125^I-Ad5wt (Fig. 5A, complete immunoblot Supplementary Fig. 6). Contrastingly, the radioactivity levels in both treatment groups was similar (Fig. 4A,B). These findings suggest that the initial tumour-associated radioactivity is a result of virus distribution in the tumour microenvironment by the “leaky” vasculature often associated with tumours. Following the accumulation of virus in the tumours, cellular uptake of both ^125^I-Adwt and ^125^I-Ad-3∆-A20T rapidly proceeds which is dependent on multiple integrins. In the case of ^125^I-Ad-3∆-A20T, the virus can enter the cell via specific binding to the αvβ6-integrin receptor, whereas αvβ3 and αvβ5 integrins which are highly expressed on Suit-2 cells, provide additional routes for cellular entry while ^125^I-Adwt enter only through αvβ3 and αvβ5 integrins. However, the higher levels of E1A-expression in tumours infected with ^125^I-Ad-3∆-A20T at 48 h after administration suggest that αvβ6-targeting play more important roles in viral spread after subsequent rounds of viral replication and cell lysis within the tumour microenvironment. Furthermore, viral replication of Ad-3∆-A20T in Suit-2 cells grown in monocultures peaked after 96 h, compared to Ad5wt which peaked at 48–72 h, producing more viral particles over a longer duration (Fig. 5B). These results verify that after the initial distribution of radioactively labelled virus, at the later stage, higher amounts of functional Ad5-3∆-A20T accumulated in tumours compared to Ad5wt. As could be expected radioactivity rapidly declined in all tissues over time, reflecting the elimination of labelled capsid proteins once the virus is de-coated and initiates new rounds of replication while the tumour-specific targeting and cell lysis is essential for efficient intra-tumoral spread over time.

### Replication of the oncolytic retargeted Ad-3∆-A20T mutant proceeds at higher levels than Ad5wt after systemic delivery

To further validate the biodistribution of radiolabelled viruses, native non-labelled Ad-3∆-A20T and Ad5wt were administered via the tail vein in Suit-2 xenografted animals at identical viral doses. Quantitative PCR revealed that both viruses reached the tumour, spleen and liver tissues at 24–48 h after systemic administration (Fig. 5C; Supplementary Fig. 5). Relative viral genome amplification was determined as fold change obtained by normalising the E1A DNA levels in tumour and spleen to the liver per animal. We detected higher levels of viral amplification in tumour tissue compared to liver at both 24 h and 48 h time points for Ad-3∆-A20T (Fig. 5D). The retargeted virus was indeed directed more towards the tumour than the liver. Conversely, higher viral amplification was detected in liver tissues in animals treated with Ad5wt than Ad-3∆-A20T. Upon comparison between both viruses, no significant differences in viral genome quantities were detected in the splenic tissues at these low doses reflecting mainly receptor-independent macrophage-mediated clearance of virus in this organ^7,32^. To conclude, Ad-3∆-A20T readily infected tumours at sufficient levels to promote viral genome amplification while Ad5wt infected hepatic tissue more readily.

## Discussion

To date, clinical efficacy with oncolytic adenoviral mutants have been reported following intratumoral administration however, the major challenge of targeting and eliminating metastatic lesions remains to be addressed. The short half-life of adenoviruses in the human circulation is attributable to rapid blood factor binding leading to immediate hepatic and macrophage mediated elimination, unfavourably decreasing the dose at distal lesions^4,6,7^. Several strategies have been used to overcome these obstacles including, capsid modifications, polymer-coating^33,34^ and delivery by mesenchymal stem cells^35^ to attenuate Factor X-binding, hepatic elimination and ablation of erythrocyte- and neutralising antibody-binding^10,19,36^. Our approach was to retarget our selective and potent oncolytic adenovirus Ad∆∆, to αvß6-expressing pancreatic cancers by introducing the high affinity A20FMDV integrin-ligand and ablate the CAR and CR-1 binding to erythrocytes, by generating Ad-3∆-A20T^12^. To investigate Ad-3∆-A20T tumor targeting efficiency and biodistribution in longitudinal imaging studies in live animals, and to avoid further genetic alterations of the highly modified virus, we established a novel protocol for radioactive labelling followed by SPECT/CT analysis. We adapted the Iodogen method for labelling of virus with Na^125^I and demonstrated the feasibility of this approach by monitoring the initial virus uptake in tumor and normal tissue in real time.

Several radioactive labeling techniques of proteins and peptides with ^125^I have been established that are routinely used to generate trace compounds with high specific activity including the Chloramine T, Iodogen and Bolton-Hunter methods^21,29,30^. While these labeling techniques rarely cause alterations of structure and function of proteins and peptides, application of the same conditions to biologically active viruses frequently result in detrimental effects to the viral life cycle with greatly reduced viral activity^22^. To date and to our knowledge, imaging of adenoviruses that have been directly radiolabeled have not been feasible. This is largely due to the irreversible modifications of the capsid proteins ensued by non-physiological reaction conditions that drastically decrease or prevent viral infection, genome amplification and gene expression. In addition, the high protein concentrations that promote efficient iodination are not achievable for adenoviruses due to the tendency of virions to aggregate at high concentrations^25,27^. Step-by step modifications of the standard Iodogen iodination conditions enabled sufficient incorporation of ^125^I into adenoviral mutants for preclinical SPECT/CT imaging while preserving viral bioactivity. The standard conditions for Iodogen-mediated oxidation of NaI (*e*.*g*. basic pH and low salt concentrations), significantly reduced viral activity, an outcome most likely caused by structural changes in the fibre and other capsid proteins preventing receptor binding and/or cellular internalization. Increasing the buffer capacity, avoiding direct exposure of virus to the oxidizing agent and prolonging the reaction time overcame these challenges resulting in functional virus. Despite lower efficiency of radioisotope incorporation with this adapted protocol, we were able to determine the initial distribution of several labeled adenoviruses in real time by SPECT/CT imaging in two PDAC xenograft models. In contrast to genetically engineered viruses such as those that express the human Na/I symporter (hNIS), requiring the modification of each mutant and delivery of ^125^I or ^131^I at high doses (*e*.*g.*^37,38^), our simpler direct labeling method does not require any genetic engineering and therefore enables for quicker results. Similarly, viral mutants expressing EGFP or RFP fluorescent proteins or Luciferase enzymes also require genetic modifications and the methods used for detection are generally less sensitive.

Our novel Ad-3∆-A20T mutant was designed to specifically target αvß6-integrin expressing PDAC tumours via systemic delivery not only to reach the deep anatomical location of the pancreas within the abdominal cavity but also to reach the widespread metastatic sites^12,39^. Interestingly, both the αvß6-integrin targeted Ad-3∆-A20T and the non-targeted Ad5wt viruses reached pancreatic subcutaneous tumours after systemic delivery in mice. It is well documented that elimination of adenoviruses takes place at hepatic Kupffer cells in a receptor-independent manner which is largely responsible for its short circulatory half-life in mice but also in humans. Human blood factors such as Factor X (FX) and IX (FIX) mediate hepatic adenovirus infection by bridging the virus to heparan sulphate proteoglycans (HSPGs) mainly on hepatocytes^5,6^. However more importantly, Ad5 hepatotoxicity is less severe in humans than in mice possibly due to the entrapping of human erythrocytes by the virus, decreasing the rate of hepatocyte uptake and Kupffer cell clearance^4^. The high affinity binding of Ad5 to CAR and CR-1 on erythrocytes in humans does not occur in the murine circulation. In our study, it is therefore likely that the apparent similar uptake-rates of Ad5wt and Ad-3∆-A20T in tumours were due to the lack of Ad5wt erythrocyte-binding since the ablation of CAR- and CR-1-binding, a “design feature” of the Ad-3∆-A20T could not be demonstrated in our murine model as such binding is obsolete in mice. Another reason for the similar immediate distribution of radioactivity to the xenografted tumours may be due to the “leaky” vasculature often present in tumours. The actions of enhanced permeability and retention (EPR) may have disguised any differences in initial receptor-mediated cellular uptake of Adwt and Ad-3∆-A20T, whilst also supporting the accumulation of both viruses to the tumour microenvironment. Furthermore, endocytosis of virus within tumour cells may not be solely dependent on αvß6-integrin binding, since Suit-2 cells express other receptors important for adenoviral binding and internalisation including CAR, αvß3- and αvß5-integrins^12^. Overall, our findings demonstrate that in mice, the initial tumour uptake of both ^125^I-labelled and non-labelled Adwt and Ad-3∆-A20T was rapid after systemic delivery. The maximum amount of the tumour-associated radiolabelled virus was reached at 4–24 h post-administration, but more importantly by 48 h, Ad-3∆-A20T replication proceeded at higher levels than Adwt. Presumably, the improved intra-tumoural spread and replication for Ad-3∆-A20T was caused by additional rounds of replication in the tumour microenvironment. In support of this assumption, Ad-3∆-A20T genomes and E1A expression were present at higher levels than Adwt by 48 h in tumours. In parallel, a decreased accumulation of Ad-3∆-A20T, reflecting the reduced genome amplification and replication compared to Adwt in liver tissue was observed. Taken together, we demonstrate that systemic delivery of the αvß6-integrin retargeted Ad-3∆-A20T virus reached subcutaneous tumour xenografts in athymic mice and propagated more efficiently within the tumours than the native Ad5wt virus.

Unfortunately, one limitation of studying adenoviruses in murine models is the lack of viral binding to murine erythrocytes and blood factors, which is a major obstacle for delivery in humans. However, we and others previously demonstrated that the modifications incorporated in Ad-3∆-A20T decreased binding to human erythrocytes and Factor IX/complement-4 binding protein (C4BP) which could result in higher circulating levels of virus in patients^6,20,40^. We expect that additional modifications of the virus may be required prior to clinical evaluation specifically, modifications of hexon to prevent the binding of neutralising antibodies and/or binding to FX^8,10^. The combination of CAR-binding ablation and specific tumour targeting are effective design strategies that can be included in future oncolytic mutants to improve delivery and efficacy in patients with metastatic solid tumours.

To conclude, our findings suggest that radioactive labelling of replicating oncolytic adenoviral mutants is an effective approach for studying adenoviruses in a preclinical setting without the need for time-consuming genetic modifications nor significantly compromising viral bioactivity. Combined with the SPECT/CT imaging vital information such as viral biodistribution, viral elimination rate and any off-target effects over time, all this can be recorded in a cost-effective, time-saving manner without affecting data quality. The imaging platform is a sensitive tool for studying experimental tumour lesions and may be useful for determining viral uptake in orthotopic tumours and otherwise inaccessible tumours in live animals. Further refinements to the method will be necessary to enable improved detection. For example, removal of unincorporated label or conjugation of virus to prelabelled prosthetic groups for higher *in vivo* stability and labelling efficiency. The strategy holds promise for efficient selection of viral modifications and mutants prior to more extensive preclinical and clinical applications.

## Materials and Methods

### Tissue culture

Human Pancreatic ductal adenocarcinoma (PDAC) cell lines Suit-2 (Cell Services, Cancer Research UK) and Panc04.03 (ATCC, LGC Standards, UK) were cultured at 37 °C and 5% CO_2_ in Dulbecco Modified Eagle’s medium (DMEM)/10% Fetal Bovine Serum (FBS)/1% penicillin and streptomycin (Penicillin 10000 units/ml, Streptomycin 10 mg/ml; P/S) (Sigma-Aldrich, MO). Cells were regularly monitored for mycoplasma, and were STR-profiled (LGC Standards and Cancer Research UK) to confirm identical profiles to those reported by the suppliers and to the original vial.

### Viruses and infections

The viruses used in the study include the previously described modified EGFP-expressing mutants generated from species C wild-type adenovirus type 5 (Ad5wt) and Ad5A20 also expressing the 20 amino acid FMDV peptide in the fibre^11,19^. Generation of Ad∆∆ (deleted in E1ACR2 and E1B19K) and Ad5-3∆-A20T (Ad∆∆; deleted in E3gp19K, ablated in CAR and CR-1 binding, and expressing the FMDV peptide) were previously described^11,12^. The viruses were produced, purified and characterised according to standard protocols^11,41^. The viral particle (vp) to infectious unit (plaque-forming units; pfu) was 10–50 vp/pfu for all viruses. All infections were performed in serum-free DMEM and 2 h later the infection-media was replaced with 10% FBS/1% P/S in DMEM.

### Radiolabelling of virus

The optimised reaction conditions were as described below. Tris-HCl buffer 200 mM pH6.8 (1 ml) was added to a precoated Iodogen (1,3,4,6-tetrachloro-3α,6α-diphenyl glycoluril) tube (ThermoFisher Scientific, UK) and removed, followed by the addition of 20–27 µl of Tris-HCl buffer (200 mM, pH6.8) and 25–33.3 µl of Na^125^I (85–123 MBq) in 0.04 M NaOH (Hartmann GmbH, Germany). After 2 min, the reaction mixture was transferred to an aliquot of 200–267 µl of virus (1.23 × 10^8^–2.62 × 10^10^ vp; 7.2 µg–7.65 µg protein) and incubated for another 2 min, 24 °C. The final reaction mixture was pH7.4-7.8. The radiolabelling efficiency was measured using iTLC-SG (Agilent, CA) with a mobile phase of 85% MeOH, and exposed on phosphor screens, detected by Cyclone (Perkin Elmer, CT) and analysed by the OptiQuant software. The percentage of free Na^125^I and virus-bound ^125^I was determined. The reaction mixture was diluted with PBS containing 0.01% BSA. Aliquots were analysed for viral integrity and function by denaturing polyacrylamide gel electrophoresis (SDS-PAGE) and radioactive viral proteins were detected on phosphor screens (Cyclone). In some studies, free ^125^I was removed by column purification (Virabind™) according to the manufacturer’s instructions (Cell Biolabs, CA) for further analysis of ^125^I-labelled virus. In these experiments, 10 µg of Tyrosine (Sigma-Aldrich) was added to the labelled sample before column purification. Identical reaction protocols using non-radioactive NaI were applied to evaluate viral infectivity and replication by flow cytometry, immunoblotting, qPCR and replication assays.

### Immunoblotting

Tumours and spleens were collected from mice and homogenised by sonication in ice-cold RIPA Buffer (50 mM Tris-Cl pH8.0, 150 mM NaCl, 1% Triton X-100, 0.5% sodium deoxycholate, 0.1% SDS) supplemented with a protease inhibitor cocktail and PhosSTOP phosphatase inhibitor (Roche Diagnostics, Switzerland). The protein concentration of each lysate sample was determined using the BCA assay as per manufacturer’s instructions (ThermoFisher Scientific). To each 20 µg of lysate sample, 2 × Laemmli buffer (0.125 M Tris-HCl pH6.8, 20% glycerol, 4% SDS, 0.01% bromophenol blue, 10% β-mercaptoethanol) was added and incubated at 95 °C for 5 min. Protein lysates were resolved by SDS-PAGE using the Bio-Rad system (Bio-Rad Laboratories Ltd, UK) and transferred onto PVDF membranes (ThermoFisher Scientific). Membranes were incubated with mouse anti-E1A (1:400; M58, GeneTex, TX), and mouse anti-vinculin (1:2000; Abcam, UK) overnight at 4 °C in PBS containing 5% non-fat milk and 0.1% Tween (PBS-T). After washing with PBS-T, membranes were incubated for 1 h at room temperature with anti-mouse HRP conjugated secondary antibodies (1:2000; Dako, UK). Protein bands were detected by enhanced chemiluminescence substrate (ECL; PerkinElmer) and visualised using digital capture (iChemi-XT imaging system; Syngene, UK). Densitometric analysis was performed using the NIH Image J software.

### Flow cytometry

Cellular infectivity was quantified by monitoring the percentage of EGFP positive cells using flow cytometry. Cells were seeded into 6-well plates (1 × 10^5^cells/well), incubated at 37 °C for 24 h prior to infection with EGFP-expressing viruses (Adwt, Ad5A20) for 2 h at 37 °C. The infection media was replaced with DMEM/10% FBS, cells were incubated for 24 or 48 h and detached with Trypsin. Detached cells were washed twice and resuspended in DMEM/0.1%BSA in preparation for flow cytometry (FACSCalibur, BD Biosciences, UK). The percentages of cells detected in the FL1 setting were compared to non-infected cells, acquiring 10,000 events per sample and analyzed using the FlowJo software 8.8.6 (Tree Star Inc.).

### *In vivo* studies

All *in vivo* experiments were performed under the approved UK Home Office Project Licence P1EE3ECB4 at Barts Cancer Institute in accordance with the Animals Scientific Procedures Act 1986.

### *In vivo* tumour growth and viral tissue distribution

Tumour cells were inoculated subcutaneously in one or both flanks of CD^*nu/nu*^athymic mice (Charles River, UK) with Suit-2 cells in sterile PBS (1 × 10^6^cells/200 µl) and Panc04.03 cells in PBS:Matrigel (Sigma-Aldrich; 1:1) (1 × 10^6^cells/200 µl). Tumour volumes were estimated twice weekly by calliper measurements: volume = (length × width^2^ × π)/6, and animals were monitored and treated according to UK Home Office Regulations. When tumours were 110 ± 30 µl animals were randomised to treatment groups and administrated the respective virus.

For viral distribution studies of non-radioactive mutants, purified viruses (Adwt, Ad-3∆-A20T) were administered via the tail vein with a single injection of 3 × 10^9^ vp in 100 µl. Tumours, spleen and liver were harvested 24, 48 and 72 h later, homogenised, and protein expression determined by immunoblotting and/or viral DNA determined by qPCR.

### *In vivo* magnetic resonance imaging (MRI)

In addition to calliper measurements of subcutaneous tumours, MRI was used to monitor subcutaneous and internal tumour spread. Mice were imaged by MRI (Bruker Icon 1T) on a heated bed to preserve body temperature (36.5–37.5 °C) and were subjected to isoflurane anaesthesia (flow rate of 1.5l/min, maintenance level of 1.5–2%). The anaesthetic level was adjusted so that the respiratory rate was approximately 30–60 breaths per minute as measured by a pressure sensitive pad. Respiratory gating was also employed to minimise movement artefacts; images were recorded between breaths. Eyes were kept hydrated with eye-gel (lubrithal 321/00/12/PUVPT, Dechra). A T2 survey scan was carried out using Paravision 6.0.1 software with a repetition time (TR) of 3377 ms and echo time (TE) of 84 ms. For each scan (4 min), 14 slices were obtained with a thickness of 1.25 mm and a slice gap of 0.25 mm. Each slice contains 110 × 110 pixels, with a resolution of 0.273 × 0.273 mm, corresponding to a field of view of 30 × 30 mm. An estimate of the tumour volume and potential internal spread was obtained by drawing 3D regions of interest (ROI) using VivoQuant software (version 3.5, InviCRO LLC, MA).

### *In vivo* Nano-SPECT/CT imaging

For viral distribution studies using radioactive viruses and SPECT/CT imaging, viral particles (Ad5wt, Ad5A20T and Ad-3∆-A20T, typically 3–8.2 × 10^9^ vp in 100 µl of iodination reaction mixture (20 MBq) were administered via the tail vein with a single injection. Thirty minutes prior to viral administration, 5 mg NaClO_4_ in 500 µl PBS was delivered intraperitoneally to each animal to block uptake of free iodine via the Na/I symporter (NIS). Animals were anesthetized with 2% Isoflurane gas in air/O_2_ (1 l/min) for whole body imaging at 0.5, 1, 4, 24, and 48 h after virus administration. All studies were performed according to UK Home Office Regulations. SPECT-imaging was performed using a NanoSPECT/CT four-head camera (Bioscan Inc, Washington D.C.) fitted with 1.4 mm pinhole collimators in helical scanning mode (24 projections, 40 min acquisition). CT images were acquired with a 45-kVP X-ray source (1500 ms exposure, 180 projections). In some studies, a multiple bed system was used to allow three mice to be imaged simultaneously with body temperatures maintained at 36.5–37.5 °C via a warm air heating system. Reconstructed images were merged (CT recon; InVivoScope Invicro LLC and SPECT recon; HiSPECT, Scivis GmbH, Göttingen, Germany) and the region of interest (ROI) was analysed using VivoQuant software (Invicro LLC). ^125^I activity in tumour, liver and spleen was quantified with 3D ROI analysis. Radioactivity in tumours, liver and spleen was corrected for % labelled virus injected in each animal and normalized to the weight of the tumour at the end of the study. For liver tissues representative fixed liver volumes were used for the 3D ROI analysis. After completion of the 48 h imaging time-points tumours, liver and spleens were collected, weighed and processed for immunoblotting, and radioactivity was quantified using a Compugamma CS counter (LKB Instruments, Australia).

### Quantitative PCR

Whole tumour, spleen and liver tissues were removed and collected from mice at 24–72 h after tail vein administration of Adwt or Ad5–3∆-A20T. Serum free media (500 µl) were added to tissue specimens and were mechanically dispersed in cell strainers (70 µM, Falcon). Samples were lysed in 200 µl lysis buffer (ATL buffer) provided in the DNA extraction kit (DNeasy Blood and Tissue kit, Qiagen). Double-stranded DNA extraction proceeded according to the manufacturer’s instructions after determining quality and quantity of harvested DNA (Nanodrop; ThermoFisher, Scientific, UK). Quantitative PCR was performed using 20 µg of extracted DNA in 20 µl reaction mixture containing Power SYBR Green Mastermix (ThermoFisher). Relative changes in viral DNA amplification was determined by the ∆∆CT method relating the DNA levels in tumour and spleen to the liver levels in each animal. The following primers were used: *E2A F* (5′-GGA TAC AGC GCC TGC ATA AAA G-3′); *E2A R* (5′-CCA ATC AGT TTT CCG GCA AGT-3′); *GAPDH F* (5′ TGG GCT ACA CTG AGC ACC AG-3′) and *GAPDH R* (*5*′ GGG TGT CGC TGT TGA AGT CA-3′). For absolute quantification of viral genomes in the same tissue specimens, a standard curve was generated using pure genomic Ad5wt DNA as template. The following primers were used: *E1A F* 5′ TGT ACC GGA GGT GAT CGA TCT 3′; *E1A R*′ TCG TCA CTG GGT GGA AAG C 3′. The thermal cycling profile were: 2 min at 50 °C, 10 min at 95 °C and 40 cycles of 15s at 95 °C and 1 min of 60 °C. Assays were performed in MicroAmp Optical 96-Well plates and Real-Time PCR system 7500 (Applied Biosystems, CA).

### Viral replication by the 50% tissue culture infectious dose (TCID_50_) assay

Suit-2 cells were seeded into 6 well plates at 1 × 10^5^ cells/well and after 24 h, cells were infected with 100 particles per cell (ppc) of Ad5-3∆-A20T. Cell culture media and cells containing virus were collected and freeze-thawed three times in liquid nitrogen at 24 h–192 h post infection. Viral samples were serially diluted in 96-well plates containing A549 cells (1 × 10^4^/well) as detector cells. Viral replication was determined as previously described for TCID_50_ assays^11,12^.

## Supplementary information

Supplementary figures 1-6
