# Supplementary material for: Systemic delivery and SPECT/CT in vivo imaging of 125I-labelled oncolytic adenoviral mutants in models of pancreatic cancer
Source: Sci Rep. 2019 Sep 6;9:12840. doi: 10.1038/s41598-019-49150-9 (PMC6731255; doi:10.1038/s41598-019-49150-9)
Supplement: Supplementary file 1 — Supplementary figures 1-6 [file 41598_2019_49150_MOESM1_ESM.pdf]

Supplementary figures 1-6 with figure legends

Systemic delivery and SPECT/CT *in vivo* imaging of ^{125}I-labelled oncolytic adenoviral mutants in models of pancreatic cancer

Y.K. Stella Man[1], Julie Foster[1], Elisabete Carapuca[1], James A. Davies[2], Alan L. Parker[2], Jane Sosabowski[1], Gunnel Halldén[1]

Supplementary Figure 1

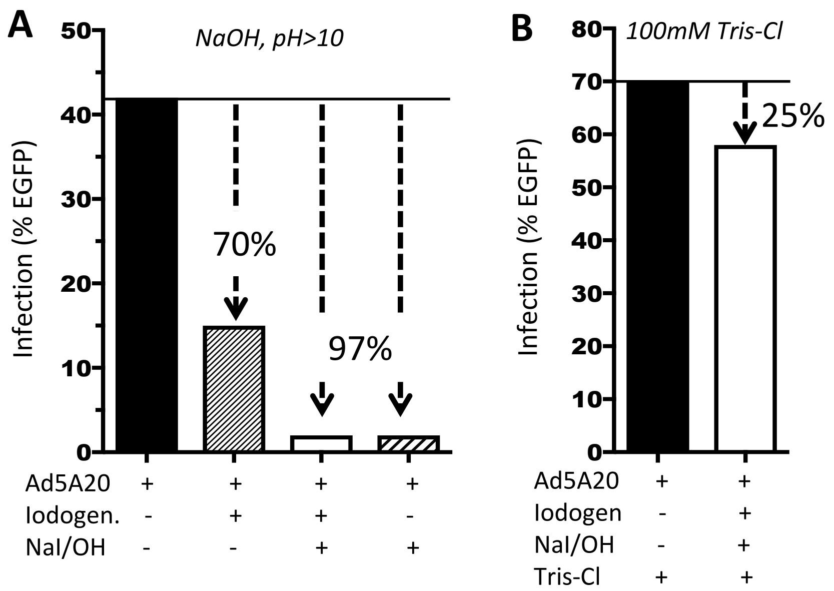

Supplementary Figure 1. Flow cytometry of Panc04.03 cells infected with non-radioactive I-Ad5A20 or unlabelled Ad5A20 after exposure to different labelling conditions. A) Up to 70% of viral infectivity was lost after exposure to Iodogen for 3min in PBS and up to 97% in the presence of NaOH 0.1M pH11 with or without Iodogen. **B)** Flow cytometry treated as in **A** with the exception that the reaction mixture was buffered by the addition of 0.1mM Tris-Cl pH4.0 to bring pH to 7.4 prior to addition of virus. Up to 75% of viral infectivity was retained under these conditions. Representative data.

Supplementary Figure 2

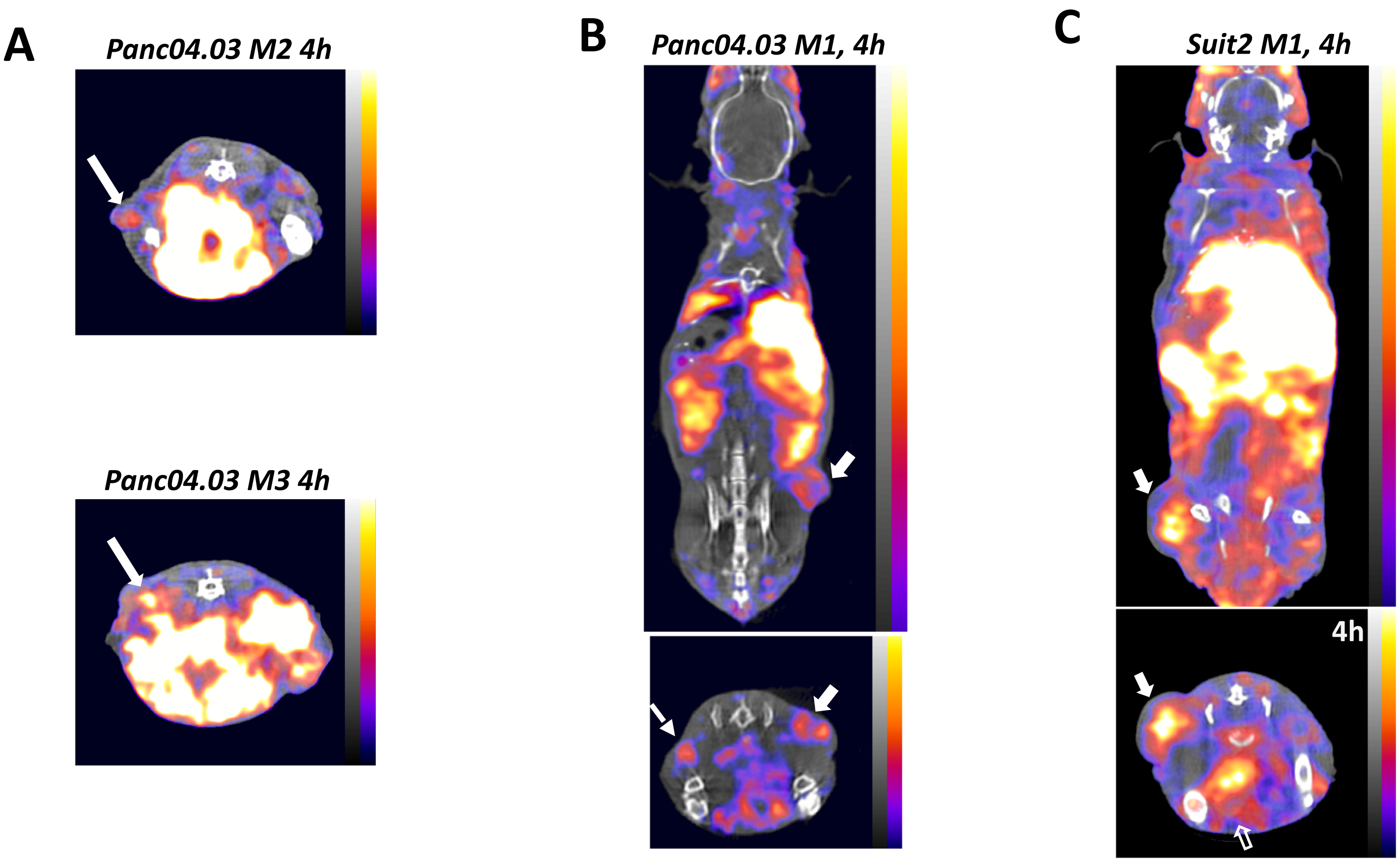

Supplementary Figure 2. ^{125}I-Ad5A20 accumulates in pancreatic tumour xenografts after tail vein injection. Intravenous administration of the αvβ6-retargeted and radioactively labelled ^{125}I-Ad5A20 in 200μl (8.2x10^{9}vp of 2.5mBq/vp) to animals with Panc04.03 and Suit-2 xenografts, imaged by SPECT/CT 4h later. **A)** Selected images are transverse views of mouse 2 and 3 (M2, M3); the subcutaneous Panc04.03 tumour is indicated by white solid arrows. **B)** Same animal as in Fig. 2B (M1), images are coronal and transverse views. The subcutaneous tumour is indicated by white solid arrow and the internal tumours by white dashed arrow. **C)** Same animal as in Fig. 2C (M1) with a subcutaneous Suit-2 xenograft tumour, imaged 4h after intravenous administration (7x10^{9}vp). Selected images are coronal and transverse views, the subcutaneous tumour is indicated by white solid arrow and the open arrow indicates the bladder. A-C) At 4h after administration the majority of the radioactivity was seen in the liver and spleen, which decreased over time. The Panc04.03 tumours grew at level close to liver and images are saturated by radioactivity in this tissue at the 4h timepoint. The scales for SPECT/CT images are 0.006 - 0.0225 kBq.

Supplementary Figure 3A

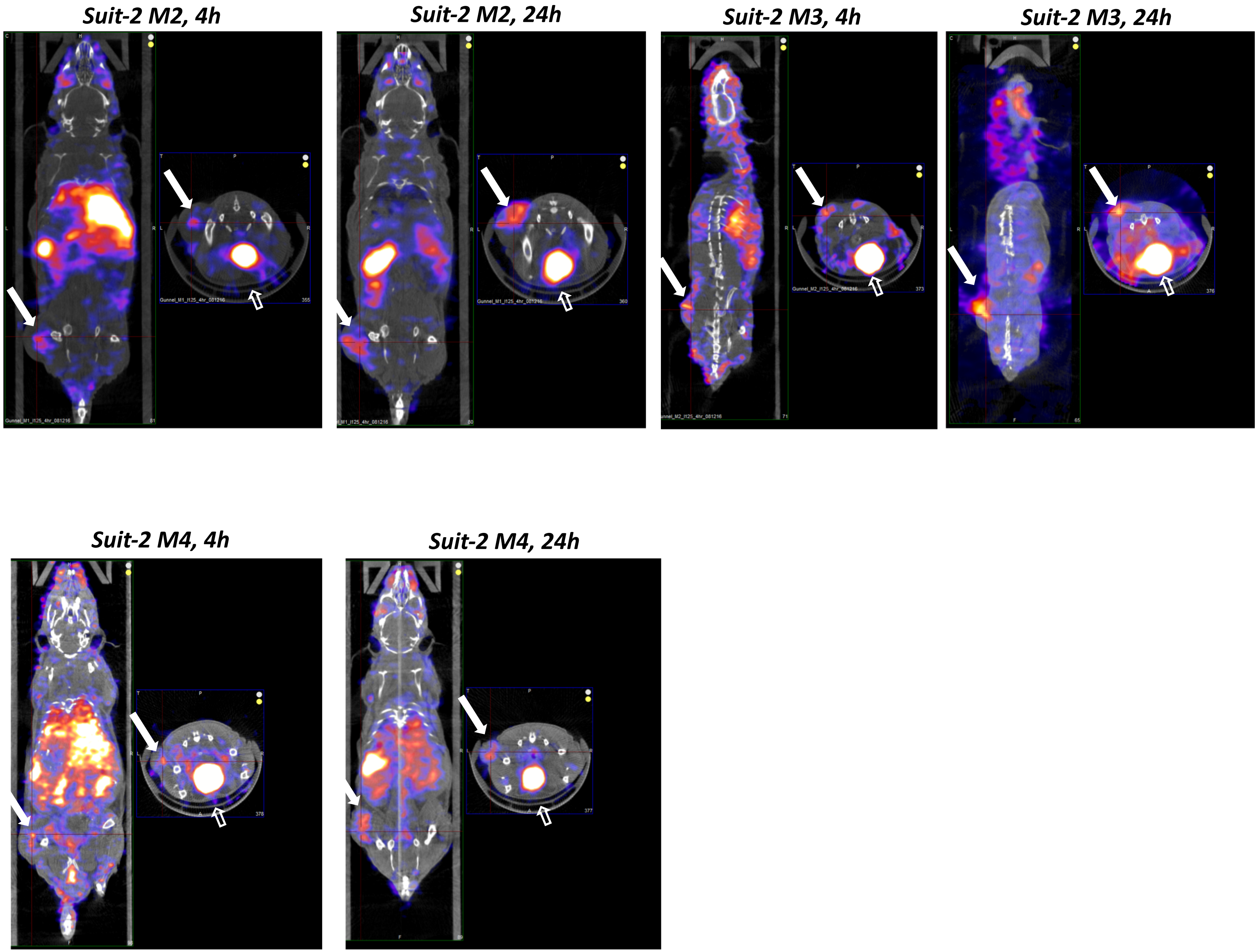

Supplementary Figure 3B

Suit-2, Ad-3Δ-A20T, M1 24h

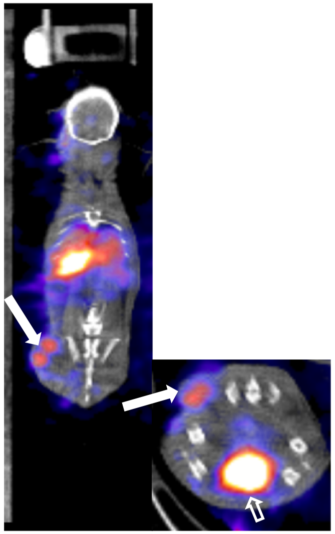

Suit-2, Ad-3Δ-A20T, M2 24h

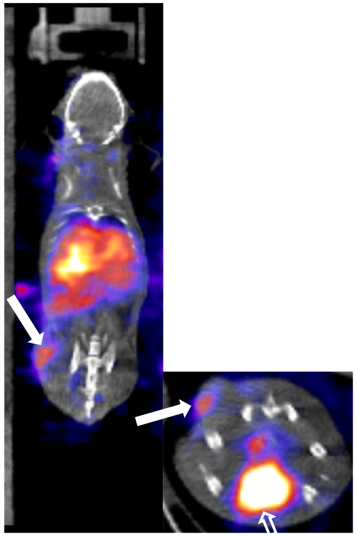

Suit-2, Ad-3Δ-A20T, M3 24h

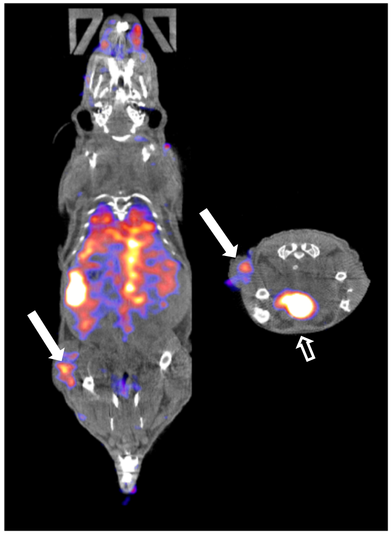

Suit-2, Ad-3Δ-A20T, M7 24h

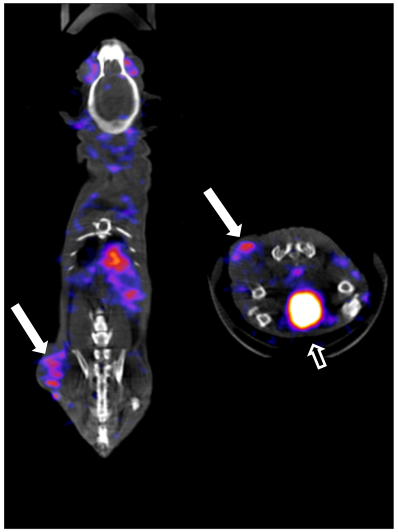

Suit-2, Adwt, M8 24h

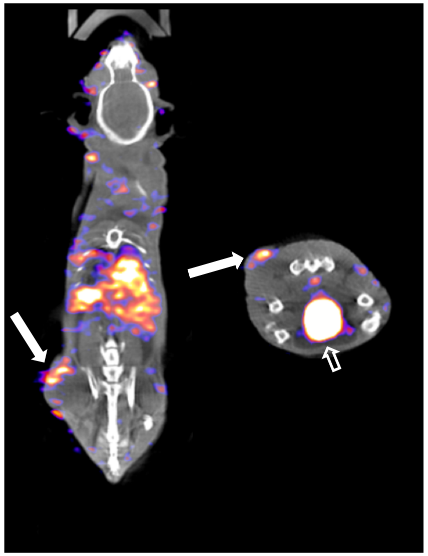

Supplementary Figure 3. ^{125}I-Ad5A20 accumulates in Suit-2 pancreatic tumour xenografts after tail vein injection, imaged by SPECT/CT. A) Three animals (M2-M4) with Suit-2 subcutaneous xenografts were administered the $\alpha v\beta 6$-retargeted, and radioactively labelled ^{125}I-Ad5A20 in 200μl (7x10^9vp of 2.5mBq/vp); 10.8MBq/animal) via the tail vein and imaged by SPECT/CT after 4h and 24h. **B)** Four animals with Suit-2 subcutaneous xenografts were administered the $\alpha v\beta 6$-retargeted, and radioactively labelled ^{125}I-Ad-3Δ-A20T (M1-3, and 7) (1.83mBq/vp in 200μl) and ^{125}I-Ad5wt (3.27mBq/vp in 200μl; M8) via the tail vein. Live animals were imaged by SPECT/CT after 24h, selected images are coronal and transverse views.; White solid arrows indicate subcutaneous tumours and the open arrow indicates the bladder.

Supplementary Figure 4

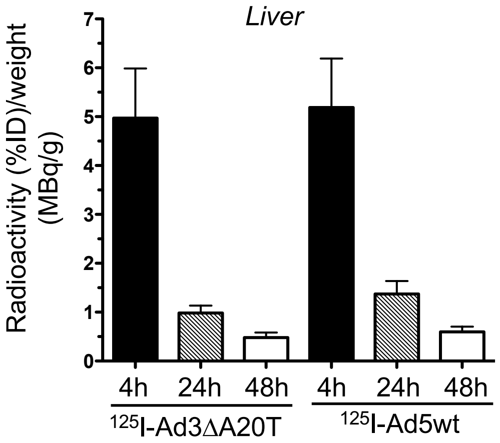

Supplementary Figure 4. Biodistribution and quantification determined by SPECT/CT imaging after tail vein administration. Tumours, spleen and liver tissues were collected 48h after treatment of animals with ^{125}I-Ad-3Δ-A20T and ^{125}I-Ad5wt (n=4 per group). Livers were weighed and correlated to the respective radioactivity SPECT/CT measurement at each time point, determined by ROI analysis using VivoQuant software. Data were corrected for injected dose (%ID) of radiolabel in tumours (left panel) (n=4/group), averages ± SD.

Supplementary Figure 5

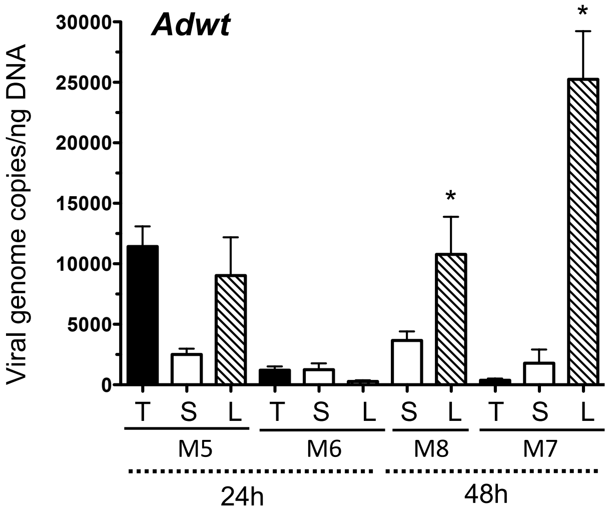

Supplementary Figure 5. Viral genome amplification in tumour, liver and spleen tissues after systemic administration of Ad5wt. Relative copy numbers of viral genomes in tissue specimens harvested from animals 24h or 48h after tail vein administration of non-labelled Adwt determined by qPCR. Values are expressed as average copy numbers of E1A per ng of DNA ± SEM in individual animal; n = 4. Tumor (T), spleen (S), and liver (L) tissues from respective animal (5-8).

Supplementary Figure 6

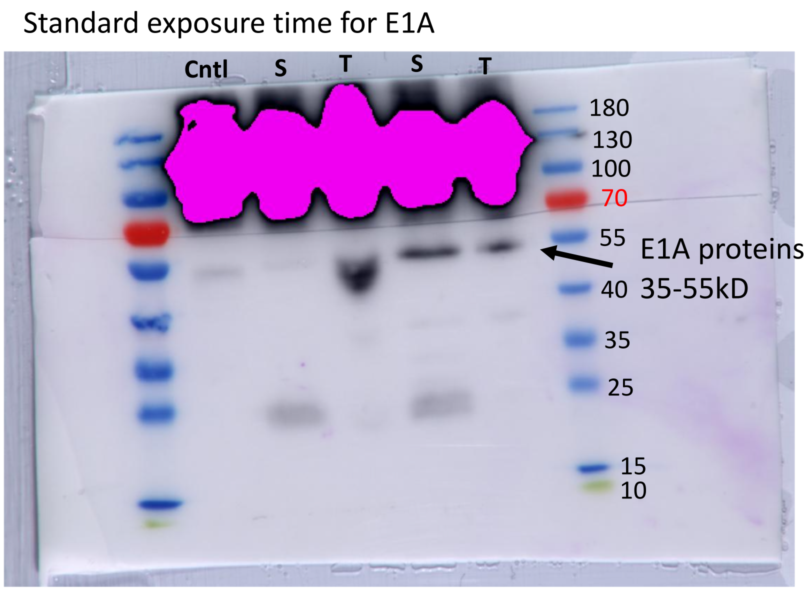

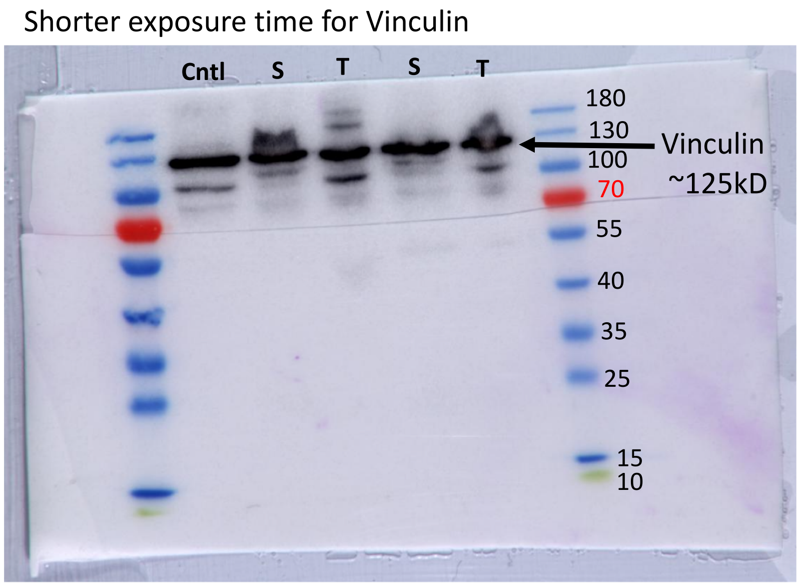

Supplementary Figure 6. Complete immunoblot represented by the cropped blots in Figure 5A. For loading of samples and processing see Figure legend 5A. Marker = ThermoScientific Page Ruler Prestained protein ladder.
